# PYRE insertion within HIV-1 subtype C p6-Gag functions as an ALIX-dependent late domain

**DOI:** 10.1038/s41598-018-27162-1

**Published:** 2018-06-11

**Authors:** Devidas Chaturbhuj, Ajit Patil, Raman Gangakhedkar

**Affiliations:** 1 HIV Drug Resistance Laboratory, National AIDS Research Institute (ICMR), Pune, 411026 Maharashtra India; 2 Symbiosis International University (SIU), Lavale, Mulshi Taluka, Pune, 412115 Maharashtra India; 3 Department of Clinical Sciences, National AIDS Research Institute (ICMR), Pune, 411026 Maharashtra India

## Abstract

ALG-2 interacting protein X (ALIX) links HIV-1 Gag to the components of ESCRT-III. HIV-1 engages the ALIX via its nucleocapsid and LYPXnL motif in p6. Overexpression of ALIX corrects the release defect of PTAP deleted HIV-1 via LYPXnL/ALIX pathway. However, HIV-1 subtype C lacks the LYPXnL motif and hence cannot employ LYPXnL/ALIX mechanism. Though the preferential occurrences of PYXE insertion in HIV-1 C p6 is predicted to restore the ALIX binding site there is no functional proof to support these observations. In this study we show that HIV-1 construct with subtype C p6 having PTAP deletion and PYRE insertion (pNL-INp6ΔPTAP/PYRE) could respond to ALIX overexpression. Notably, conserved Phenyl alanine residue (F676) in ALIX was critical for ALIX mediated release of pNL-INp6ΔPTAP/PYRE implying the critical role of this hydrophobic patch in ALIX recruitment. In addition, we show that Nedd4-1 could also correct the release defect of pNL-INp6ΔPTAP/PYRE. Moreover, Nedd4-1 was more robust compared to ALIX in its ability to stimulate the release of pNL-INp6ΔPTAP/PYRE. Replication kinetic data highlights the positive effect of PYRE insertion on virus replication. In summary, our data reveals the functional role of PYRE insertion towards the cooperative mechanism of ALIX/Nedd4-1 in virus release in the absence of PTAP/Tsg101 pathway.

## Introduction

HIV-1 Gag harbours a short 52 amino acid peptide p6 at its C-terminus that plays a vital role in virus release form host cell membrane. Two types of motifs have been characterized thus far in case of HIV-1 p6: PT/SAP and LYPXnL^1–3^. PTAP motif engages the cellular Tsg101 while LYPXnL is known to interact with ALG-2 interacting protein-X (ALIX)^4,5^. An interaction with Tsg101 and ALIX links HIV-1 Gag to host cellular fission machinery [Endosomal Sorting Complex Required for Transport (ESCRT)] thus facilitating viral release^6–8^. ALIX bro1 domain engages the HIV-1 Gag nucleocapsid while LYPXnL motif in p6 is the docking site for ALIX V domain^9–11^. Interaction with ALIX links HIV-1 Gag to components of ESCRT-III machinery. Mutations in the LYPXnL motif abrogate the interaction between HIV-1 p6 Gag and ALIX V domain^12–14^. Loss of this interaction eventually hampers the ALIX mediated virus release. HIV-1 lacking the PTAP motif employs ALIX mediated release and an intact LYPXnL motif is the necessity for this effect to take place^12,15^. Subtype C HIV-1 lacks the consensus LYPXnL motif and hence do not possess the ability to engage ALIX V domain for ALIX mediated release^16^.

There have been reports of occurrence of a novel tetrapeptide insertion PYXE [where X is any amino acid out of arginine (R), Lysine (K), or glutamine (Q)] in Ethiopian subtype C HIV-1 p6 sequences derived from therapy naïve patients^17^. In case of Indian and South African HIV-1 subtype C, PYXE insertion has been associated with treatment failure^17,18^. Introduction of PYXE insertion is predicted to restore the ALIX binding site in subtype C p6. However, the functional characterization of this insertion towards ALIX mediated virus release in subtype C has not been addressed. Zhai *et al*. have demonstrated that SREKPYKEVTEDLLHLNSLF sequence in SIV_mac239_ functions as an ALIX recruiting site^19^. PYXE insertion in HIV-1 subtype C introduces the stretch of a sequence similar to ALIX binding motif characterized in SIV_mac239_. SREKPYKEVTEDLLHLNSLF sequence in SIV is dubbed as Type 3 ALIX binding motif. Type 1 (LYPDL) and Type 2 (LYPLT/ASL) ALIX binding motifs are present in equine infectious anaemia virus (EIAV) and HIV-1 respectively. Canonical type 2 ALIX binding motif (LYPXnL) is predominant in the HIV-1 strains. While HIV-1 subtype C lacks consensus LYPXnL motif and PYXE insertion introduces the elements similar to the type 3 ALIX binding motif in HIV-1 C p6, we wondered whether this could restore the ALIX driven release mechanism in these viruses. In present study we have used the PYRE, a variant of PYXE to investigate its role towards ALIX mediated release in HIV-1 subtype C.

## Results

### Effect of PYRE insertion on ALIX mediated virus release

HIV-1 subtype C p6 lacks the critical LYPXnL consensus sequence required to recruit the ALG-2 interacting protein X (ALIX). Due to lack of L35Y36 residues HIV-1 subtype C p6 cannot interact with ALIX and hence it does not support the ALIX mediated release^16^. Neogi *et al*. reported a novel PYXE insertion in HIV-1 subtype C and the same is predicted to restore the recruitment of ALIX by HIV-1 C p6 protein^17^. Unfortunately there is no data available on the functional consequences of this insertion to support the ALIX mediated virus release.

**Figure 1:**
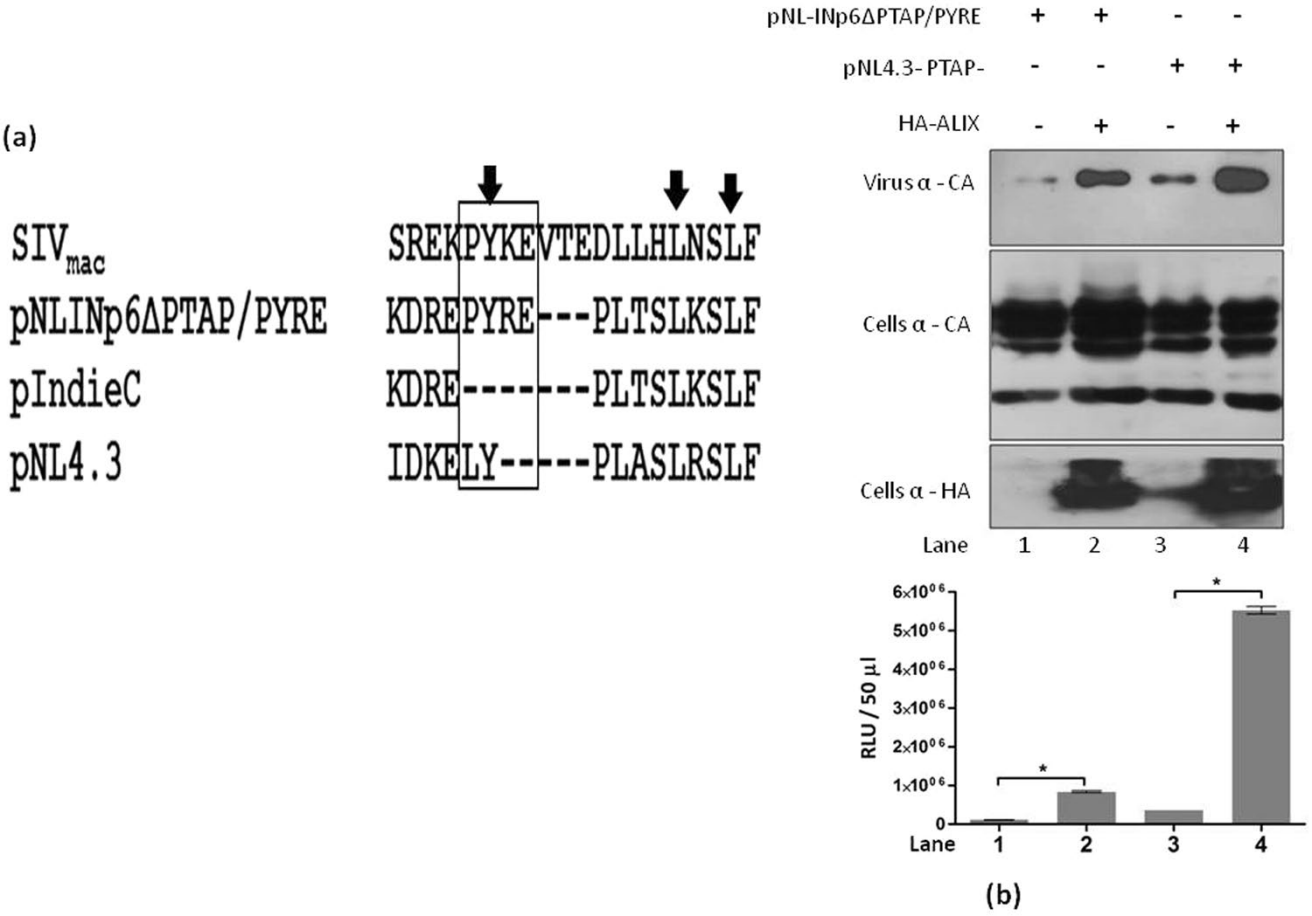
(**a**) Alignment of pNL-INp6ΔPTAP/PYRE P6 with SIVmac, pNL4.3 (Subtype B) and pIndie C1 (Subtype C) P6 proteins. PYRE insertion in pNL-INp6ΔPTAP/PYRE is boxed with respective SIVmac type 3 ALIX binding motif and HIV-1 LYPXnL motif. PYRE insertion in HIV-1 subtype C P6 incorporates the amino acid residues similar to type 3 ALIX binding motif. Functionally important ALIX binding sites in SIVmac are marked with down arrow. Canonical ALIX binding motifs are missing in pIndie C1 p6 (Subtype C). (**b**) ALIX overexpression rescues pNL-INp6ΔPTAP/PYRE release. 293T cells were transfected with 1 µg of pNL-INp6ΔPTAP/PYRE (lane 1) and pNL4.3PTAP- (lane 3) alone or with 3 µg HA-ALIX respectively (lane 2 and 4) Western blots showing virus production (top gel panel), cellular Gag protein (Middle gel panel) and exogenous ALIX expression (bottom gel panel). Graph below shows the infectivity of released virions in TZM-bl single cycle infectivity assay (n = 3 ± standard deviation). *P* value were determined using a student t test. *p < 0.001.

In a study published by our group, we had reported that pNL4.3 carrying PTAP deleted subtype C p6 could not respond to the ALIX overexpression^16^. To address the functional consequences of PYRE insertion with respect to ALIX mediated virus release we constructed a chimeric pNL4.3 expressing PTAP deleted pIndie C1 p6 with PYRE insertion (pNL-INp6ΔPTAP/PYRE). As shown in the Fig. 1a PYRE insertion introduces amino acid residues functionally equivalent to the ALIX binding motif characterized in SIV_mac_. Plasmid pNL-INp6ΔPTAP/PYRE was transfected in to 293T cells with or without HA-ALIX. PTAP deleted HIV-1 molecular clone pNL4.3 PTAP- was also transfected with or without HA-ALIX as a control. As expected absence of a PTAP motif severely impaired the release of pNL-INp6ΔPTAP/PYRE and pNL4.3 PTAP- without affecting the cellular gag expression. Over expression of ALIX indeed stimulated the release and infectivity of pNL4.3 PTAP- (Fig. 1b compare lane 3 and 4) as expected. The interesting observation is that the over expression of ALIX also stimulated the release and infectivity of pNL-INp6ΔPTAP/PYRE (Fig. 1b compare lane 1 and 2). However, the effect of ALIX overexpression was more robust in case of pNL4.3 PTAP- compared to pNL-INp6ΔPTAP/PYRE. These experiments clearly demonstrate that HIV-1 C p6 late domain with PYRE insertion could respond to ALIX overexpression suggesting restoration of ALIX binding site.

### ALIX mediated release of pNL-INp6ΔPTAP/PYRE requires the conserved F676 residue located in the ALIX V domain

**Figure 2:**
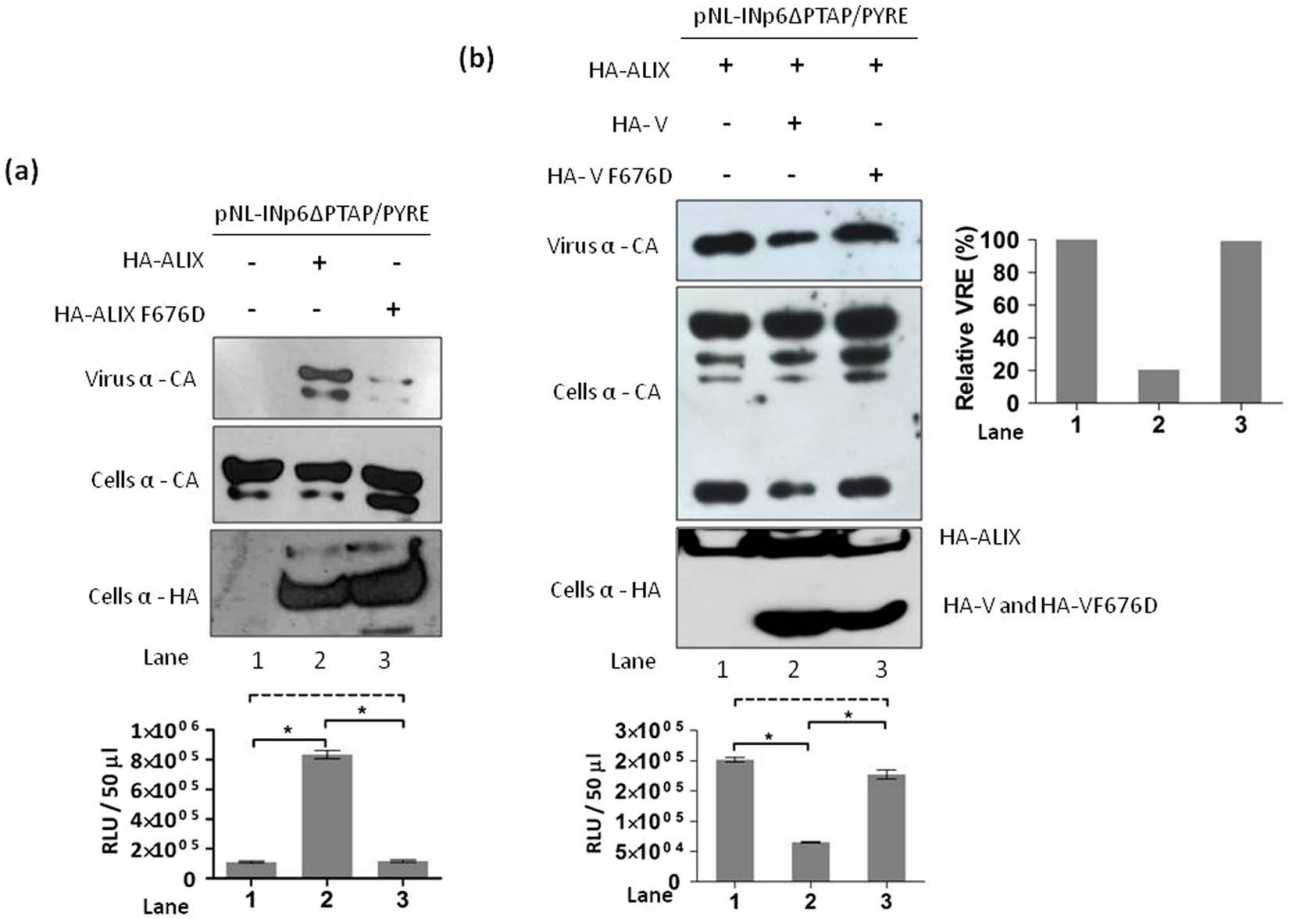
(**a**) ALIX Phe-676 hydrophobic pocket is required for pNL-INp6ΔPTAP/PYRE release. 293T cells were transfected with 1 µg pNL-INp6ΔPTAP/PYREalone (lane 1) or with 3 µg HA-ALIX (lane 2) and with 3 µg HA-ALIX F676D (lane 3). Western blots showing virus production (top gel panel), cellular Gag protein (Middle gel panel) and exogenous HA-ALIX and HA-ALIX F676D expression (bottom gel panel). Graph below shows the infectivity of released virions in TZM-bl single cycle infectivity assay (n = 3 ± standard deviation). *P* value were determined using a student t test. *p < 0.001; dashed line, no significant difference. (**b**) Overexpression of HA-V but not HA-V F676D inhibits the ALIX mediated release of pNL-INp6ΔPTAP/PYRE. 293T cells were transfected with 1 µg of pNL-INp6ΔPTAP/PYRE plus 3 µg HA-ALIX (lane 1) or with 3 µg HA-ALIX plus 2 µg of HA-V (lane 2) and with 3 µg HA-ALIX plus 2 µg HA-V F676D (lane 3). Western blots showing virus production (top gel panel), cellular Gag protein (Middle gel panel) and exogenous HA-ALIX, HA- V and HA- V F676D expression (bottom gel panel). Graph below shows the infectivity of released virions in TZM-bl single cycle infectivity assay (n = 3 ± standard deviation). *P* value were determined using a student t test. *p < 0.001; dashed line, no significant difference. The virus release efficiency (VRE) was calculated as the amount of virion-associated p24 as a fraction of the total amount (cell- plus virion-associated) of Gag relative to pNL-INp6ΔPTAP/PYREplus HA-ALIX (lane 1), which was arbitrarily set at 100.

Phenyl alanine (F676) hydrophobic pocket in ALIX V domain is critical for interaction between ALIX V domain and HIV-1 p6 LYPXnL motif. Mutant ALIX-F676D abrogates this interaction and consequently fails to rescue HIV-1 PTAP- release and infectivity^11,12,20^. As ALIX could stimulate the release of pNL-INp6ΔPTAP/PYRE we wished to study the effect of ALIX-F676D mutant overexpression on pNL-INp6ΔPTAP/PYRE release. To address this, pNL-INp6ΔPTAP/PYRE was transfected either alone and with HA-ALIX or HA-ALIX-F676D in to 293T cells. Virus release was measured by western blotting and measuring the titre of released virions on TZM-bl cells. As shown in the Fig. 2a lane 2 overexpression of ALIX could stimulate the virus release and infectivity. However, even though the cellular expression levels of ALIX and ALIX-F676D were similar, ability of ALIX-F676D to rescue the release and infectivity of pNL-INp6ΔPTAP/PYRE was reduced drastically (Fig. 2a compare lane 2 and 3).

As described earlier overexpression of ALIX V domain (fragment 364–716) acts as a dominant negative inhibitor of HIV-1 release by interacting with LYPXnL motif^20,21^. We reasoned that, if PYRE insertion is able to recruit the ALIX V domain, then overexpression of isolated V domain (ALIX region 364–716) may act as a dominant negative fragment and would hamper the ALIX mediated pNL-INp6ΔPTAP/PYRE release. Moreover, introduction of F676D mutation in isolated V domain may reverse this dominant negative effect. To check this hypothesis, pNL-INp6ΔPTAP/PYRE was transfected in to 293T cells with HA-ALIX and also with ALIX plus HA-V or ALIX plus HA-VF676D. Overexpression of HA-V lead to the robust reduction in virus release as observed by western blotting and infectivity data (Fig. 2b compare lane 1 and 2). Furthermore, we also observed that the overexpression of HA-VF676D did not inhibit the virus release and infectivity (Fig. 2b lane 3) indicating that F676D mutation reversed the inhibitory effect of V domain on ALIX mediated release. These data demonstrate that the conserved F676 residue in ALIX V domain is critical for ALIX mediated release of pNL-INp6ΔPTAP/PYRE.

### Overexpression of Nedd4-1 ubiquitin ligase corrects the pNL-INp6ΔPTAP/PYRE release

**Figure 3:**
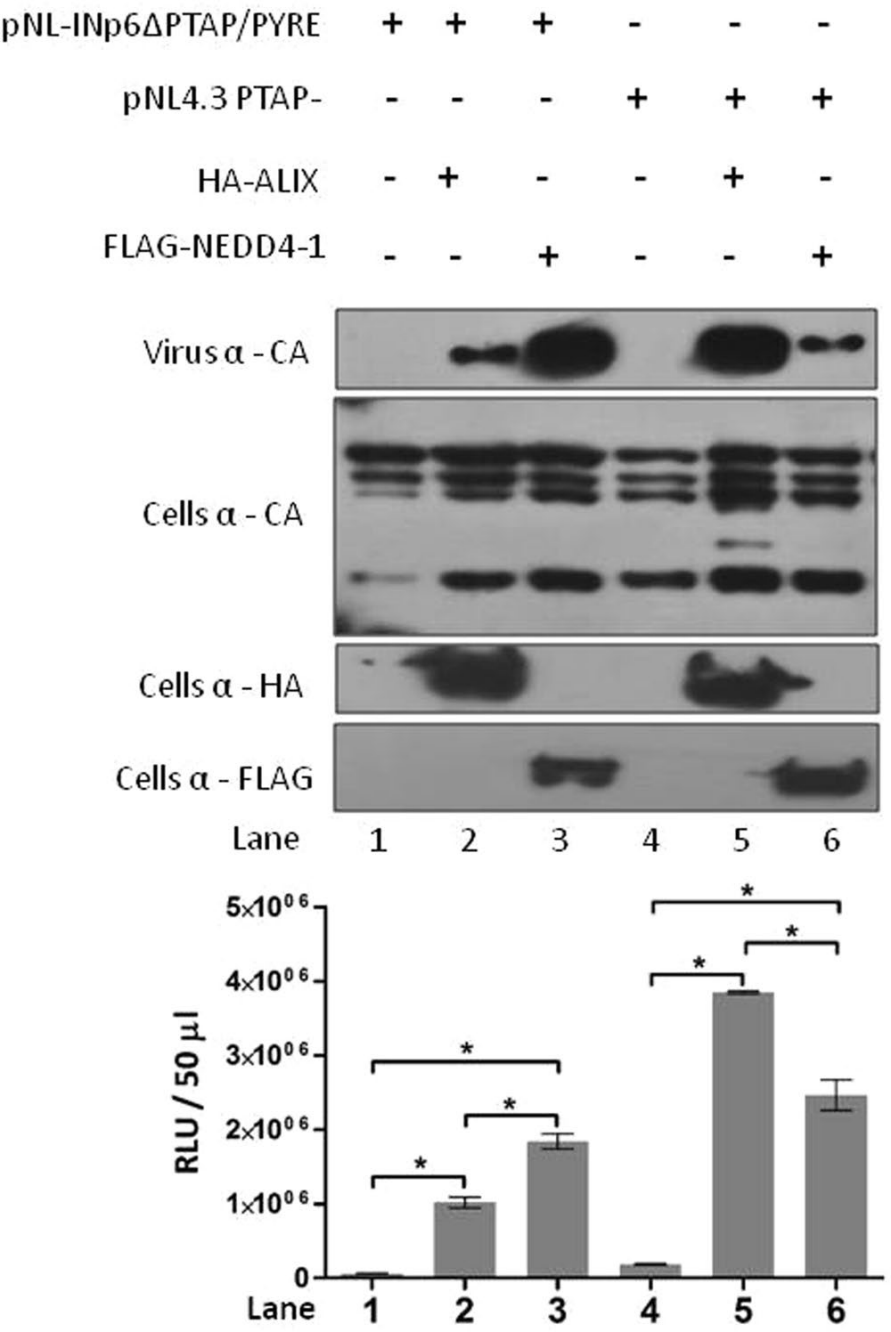
NEDD4-1 overexpression rescuespNL-INp6ΔPTAP/PYRE release. 293T Cells were transfected with pNL-INp6ΔPTAP/PYRE (lane 1) or pNL4.3PTAP- (lane 4) alone or with HA-ALIX (lane 2 and 5) and FLAG-Nedd4-1 (lane 3 and 6) respectively. Western blots showing virus production (top gel panel), cellular Gag protein (second gel panel) and exogenous HA-ALIX, FLAG-NEDD4.1 expression (gel panel 3 and 4 respectively). Graph below shows the infectivity of released virions in TZM-bl single cycle infectivity assay (n = 3 ± standard deviation). *P* value were determined using a student t test. *p < 0.001.

Nedd4-1 ubiquitin ligase has been shown to correct the HIV-1 PTAP- release defect^22,23^. Availability of the ALIX binding site is necessary for Nedd4-1 mediated release^23^. This observation led us to hypothesize that, since pNL-INp6ΔPTAP/PYRE release defect was corrected by the ALIX, Nedd4-1 overexpression may also impart the similar effect. To test this, pNL-INp6ΔPTAP/PYRE alone and with FLAG-Nedd4-1was transfected in to the 293T cells, pN4.3 PTAP- was used as a control. Western blot analysis and single cycle infectivity of released virions revealed that FLAG-Nedd4-1 was able to restore the release of pNL-INp6ΔPTAP/PYRE (Fig. 3 compare lane 1 and 3). This data clearly indicates that the Nedd4-1 is able to correct the pNL-INp6ΔPTAP/PYRE release defect. Interestingly we observed that Nedd4-1 overexpression stimulated the virus release more efficiently compared to ALIX in case of pNL-INp6ΔPTAP/PYRE.

### PYRE insertion enhances virus replication in case of multiple rounds of infection

**Figure 4:**
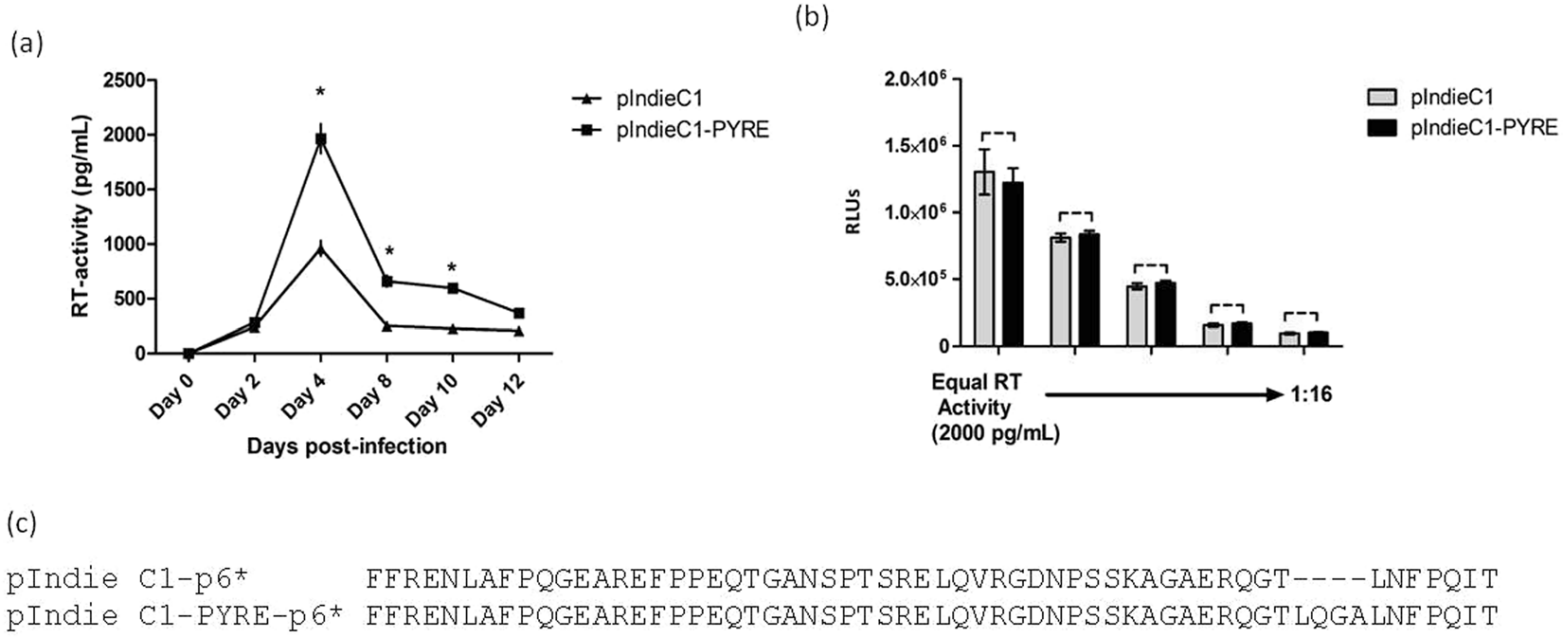
Replication Fitness of pIndieC1-PYRE versus pIndieC1. (**a**) Multiple cycle Replication capacity assay in CEM-CCR5 cells. pIndieC1- PYRE and pIndieC1 virus stock were normalised for RT activity, and used to infect CEM-CCR5 cellsseparately. Virus replication was monitored by quantifying RT-activity in the culture supernatant (n = 3 ± standard deviation). *P* value were determined using a student t test. *p < 0.001. (**b**) Single-cycle infectivity assay. pIndieC1- PYRE and pIndieC1 virus stock were normalised for RT activity, and used to infect TZM-bl cells in 96 well plates. Luciferase activity was measured two days post infection (n = 3 ± standard deviation). *P* value were determined using a student t test. *p < 0.001; dashed line, no significant difference. (**c**) Figure showing the amino acid changes in an overlapping p6-pol (p6*) reading frame due to PYRE insertion in p6-Gag.

Further we tested the effect of PYRE insertion on HIV-1 subtype C replication properties. Prior study on this aspect indicated increased replication capacity of viruses carrying PYXE insertion^24^. However, in this study the viruses generated were constructed by cloning a patient derived fragment spanning p2-INT region (p2-NCp7/p1/p6/pol-PR/RT/IN) in to the pNL4.3 background. To avoid the possibility of any differences other than PYRE insertion influencing the replication properties we introduced the PYRE insertion in HIV-1 subtype C molecular clone pIndie C1 (pIndie C1-PYRE). To evaluate the effect of PYRE insertion on viral replication pIndie C1 and pIndie C1-PYRE viruses generated in 293T cells were normalised for RT-activity values. Equal amounts of RT-activity were used to infect the CEM-CCR5 cells. Virus replication was monitored by measuring the RT-activity in medium over time. As shown in the Fig. 4a pIndie C1-PYRE showed an enhanced replication compared to pIndie C1. This finding exhibit that introduction of PYRE insertion certainly imparted the positive effect on replicative properties of pIndie C1.

There have been reports of ALIX binding site mutants Y36A, Y36S/L44R moderately (approx. 2folds) inhibiting the single cycle infectivity of HIV-1^14^. As PYRE insertion restores the ALIX binding site we asked, whether this insertion provides any advantage to the virus towards its single round infectivity also? To answer this, equal amount of RT-activity of pIndie C1 and PIndie C1- PYRE were used to infect the TZM-bl cells and the infectivity was measured by luciferase assay after 48 hrs. As shown in Fig. 4b there was no substantial effect of PYRE insertion on single round infectivity of pIndie C1-PYRE compared to pIndie C1.

## Discussion

HIV-1 subtype B p6 contains the LYPXnL motif, a type 2 ALIX binding site which is critical for Tsg101 binding site mutant virus release. However the same consensus motif is not available in HIV-1 subtype C. In our previous study we have reported that the natural deletion of L35Y36 in p6 gag eliminates the LYPXnL/ALIX auxiliary release pathway in HIV-1 subtype C^16^. In the present study we have characterized the ability of HIV-1 subtype C p6 late domain with PYRE insertion to respond to the ALIX mediated release. Introduction of PYRE sequence in HIV-1 subtype C p6 incorporates the residues similar to the type 3 ALIX binding motif characterized in SIVmac (SREK**PYKE**VTEDLLHLNSLF). This provides the HIV-1 subtype C late domain with structurally equivalent residues, which are known to participate in ALIX recruitment. Despite the sequence similarity with type 3 ALIX binding motif, functional importance of this sequence has not been established in case of HIV-1 subtype C. We observed that ALIX overexpression rescued the release defect of the PTAP deleted HIV-1 construct expressing the subtype C p6 late domain with PYRE insertion (pNL-INp6ΔPTAP/PYRE). However, in case of pNL4.3 PTAP- ALIX mediated release was more robust compared to pNL-INp6ΔPTAP/PYRE. This could possibly indicate that with respect to HIV-1, LYPXnL motif is better equipped than PYRE insertion towards ALIX mediated viral release. Never the less it is important to note that ALIX overexpression could stimulate the release of pNL-INp6ΔPTAP/PYRE.

Functional importance of ALIX V domain F676 hydrophobic patch and surrounding region is very well established in case of HIV-1 LYPXnL motif engagement^12,20^. Similarly ALIX dependent late domain activity of type 3 ALIX binding domain in SIVmac depends on the integrity of F676 pocket^19^. We also observed that ALIX-F676D lost its ability drastically to stimulate the release of pNL-INp6ΔPTAP/PYRE reinforcing the importance of this residue in ALIX recruitment and virus release. Further, Introduction of F676D mutation in isolated ALIX V domain reversed its dominant negative effect on ALIX mediated virus release. Together with the ALIX rescue experiments and ALIX-F676D mutant’s inability to restore the viral release it could be stated that the HIV-1 C p6 with PYRE insertion was able to engage ALIX and this engagement could stimulate the virus release in the absence of PTAP motif. Indeed the integrity of the hydrophobic pocket present on the long arm of ALIX V domain is essential for this effect to take place.

Ubiquitin conjugation activity of Nedd4-1 remedies the release defect of HIV-1 PTAP- in conjunction with ALIX. Presence of intact ALIX binding site in HIV-1 p6 late domain is necessary for Nedd4-1 activity^23^. We observed that Nedd4-1 is also able to restore the release defect of pNL-INp6ΔPTAP/PYRE. Nedd4-1′s ability to correct the virus budding defect is reported to be less robust compared to ALIX and Nedd4-2^22,23^. In our experiments we observed an interesting fact that Nedd4-1 was far more robust compared to ALIX towards its ability to stimulate the pNL-INp6ΔPTAP/PYRE release. These observations reflects certain underlying differential requirements of LYPXnL and PYRE motifs towards engagement of ALIX and Nedd4-1 ubiquitin ligase in the process of correcting virus budding defects. Overall these findings suggest that introduction of PYRE sequence in HIV-1 subtype C p6 late domain enables these viruses to engage the cooperative mechanism of ALIX/Nedd4-1 for virus release in the absence of primary Tsg101/PTAP mechanism.

Kiguoya *et al*. recently demonstrated that the absence of ALIX binding L35, Y36 residues from HIV-1 C sequences directly contributed to its reduced replication capacity compared to subtype B^25^. The obvious question was that while PYRE insertion restores the ALIX binding site, how it would contribute to viral replication capacity? Our replication kinetics data showed that PYRE insertion enhanced the virus replication in case of multiple round infection experiment. On the contrary we did not observe any effect of it on single round infectivity. These observations highlight that PYRE insertion may persuade its effect during the late stages of viral life cycle rather than the early events.

Moreover, PYRE insertion in p6 region also contributes to the changes in the overlapping p6-pol (p6*) open reading frame. As shown in the Fig. 4c PYRE insertion in pIndie C1 p6 leads to the simultaneous insertion in an overlapping p6* open reading frame. HIV-1 p6* plays an important role in protease activation/virus maturation. C-terminal region in p6* is known to modulate the virus maturation by regulating the process of protease folding and preventing the premature protease activation^26,27^. Dautin *et al*. in their study have credited the p6-pol to compensate for the folding defects in protease arising from the mutations within the protease reading frame^28^. In the same context more frequent occurrence of PYXE insertion in patients failing antiretroviral therapy as reported by Neogi *et al*.^17,18^ may signify the role of this insertion in complementing protease function hampered due to drug resistance mutations. Ultimately, it would be interesting to address the effect of this insertion with regard to p6* and its subsequent effect with respect to drug resistance mutations especially in protease.

In summary, our study highlights the functional attributes of PYRE insertion in HIV-1 subtype C p6 late domain with respect to auxiliary ALIX mediated virus release. As per our observations subtype C p6 late domain lacking the PTAP motif and having the PYRE insertion could respond to ALIX and Nedd4-1 overexpression. To the best of our knowledge, this is the first report highlighting the functional characterization of PYRE insertion in HIV-1 subtype C late domain, however, our study is limited to the PYRE insertion, and it would be interesting to test the role of other variants like PYKE, PYQE. Also we have inserted PYRE in an Indian subtype C background, studying the effect of these insertions in other HIV-1 strains like East African subtype C, where it is reported to occur more frequently may provide an additional information. As ALIX mediated release pathway is not available by default in HIV-1 subtype C, PYRE insertion may allow these viruses to employ the ESCRT-III components via ALIX. The questions that needs to be answered are (a) why this insertion is being selected by these viruses and (b) how does engagement of ALIX/ESCRT-III benefits them? Certainly further studies are warranted in the backdrop of (a) reports on frequent occurrence of PYXE in HIV-1 subtype C and (b) an observed contribution of this insertion in re-establishing ALIX mediated release along with replicative benefit.

## Methods

### Proviral constructs

HIV-1 molecular clone pNL4.3 PTAP- was a kind gift from Dr. Eric O. Freed, NCI, NIH, USA. HIV-1 molecular clone pIndie C1 was used as a subtype C clone^29^. HIV-1 proviral construct pNL-INp6ΔPTAP/PYRE, a chimeric pNL4.3 expressing PTAPdeleted (PTAP to LIRL) pIndie C1 p6 with PYRE insertion was constructed using Gibson assembly cloning kit (New England BioLabs Inc.): briefly, a PTAP to LIRL mutation along with PYRE insertion was introduced in to the subtype C molecular clone pIndie C1 fragment spanning 1826 to 2855 (HXB2 coordinates) by PCR mutagenesis. Further this fragment with PTAP to LIRL change and PYRE insertion was cloned in to the pNL4.3 digested with ApaI and SbfI using Gibson assembly cloning kit as per manufacturer’s protocol. Thus, the resulting construct (pNL-INp6ΔPTAP/PYRE) possesses the Nucleocapsid (p7) and p6 derived from a HIV-1 subtype C molecular clone (pIndie C1).

HIV-1 proviral construct pIndie C1-PYRE, a pIndie C1 derivative carrying PYRE insertion in its p6 late domain was constructed using Gibson assembly cloning kit (New England BioLabs Inc.). Briefly, PYRE insertion was introduced in to the p6 region of pIndie C1 using following primers, PYRE_Insertion_F 5′-CCGAAAGACAGGGAA**CCCTACAGGGAG**CCCTTAACT-3′, PYRE_Insertion_R 5′-AGTTAAGGGCTCCCTGTAGGGTTCCCTGTCTTTCGG-3′ and primers IndieC_2000_to_2027_F 5′-ATTGCAGGGCCCCTAGGAAAAAAGGCTG-3′, IndieC_p6_R 5′-TTGAGACAAGAGGTCGCTGCC-3′ were used for joining the mutant fragments by an overlap extension PCR method. The vector portion of pIndie C1 was amplified with the help of Platinum SuperFi DNA polymerase (Thermo Fisher Scientific) using primers IndieC_Vector_F 5′-GGCAGCGACCTCTTGTCTCAA-3′ and IndieC_Vector_R 5′-CAGCCTTTTTTCCTAGGGGCCCTGCAAT-3′. The insert and vector with overlapping regions at 5′ and 3′ ends were used for ligation using Gibson assembly cloning kit as per manufacturer’s protocol.

### Plasmids

Plasmid pHM6 HA-ALIX and FLAG-Nedd4-1 were kindly provided by Dr. Fadila Bouamr, NIAID, NIH, USA. HA-ALIX F676D was constructed by introducing the F676D mutation in pHM6 HA-ALIX by PCR mutagenesis. HA-V expressing ALIX V domain fragment (364–716) was constructed as described previously^16^. HA-V F676D was constructed by introducing the F676D mutation at respective position in HA-V by PCR mutagenesis.

### Virus Release Assay

293T cells (ACC-CRL-3216) were seeded at the density of 4 × 10^5^ cells per well of 6 well plate. Cells were transfected next day using calcium phosphate precipitation method (Promega Inc.) following manufacturer’s recommendations. 48 hrs after transfection culture supernatants were cleared by centrifugation and passing through 0.45 µm syringe filter. Virus was pelleted by layering 1 ml of cleared culture supernatant on 250 µl of 20% sucrose cushion and centrifugedat 25000x g for 90 min at 4 °C. Pelleted virions were dissolved in 60 µl of 2× SDS sample reducingbuffer. For cell lysates, cells were washed once with cold PBS while on ice and lysed using a cell lysis buffer [50 mM Tris. HCl (pH 7.5),120 mM NaCl, 1% Triton X-100 and protease inhibitor cocktail (SIGMA Inc.)]. Pelleted virions and cell lysates were analysed by SDS-PAGE and Western blotting using anti HIV-1 p24 monoclonal antibody (Clone 183-H12-5C, NIH AIDS Reagents Program, Division of AIDS, NIAID, NIH). Protein expression for HA-ALIX, HA-ALIX F676D, HA-V and HA-V F676D were detected using monoclonal anti-HA-peroxidase antibody (SIGMA) at 1:6000 dilution. FLAG-Nedd4-1 expression was detected using Anti-FLAG M2 monoclonal antibody (SIGMA) at 1:2000 dilution followed by anti-mouse IgG HRP 1:3000 (Thermo Fisher Scientific). Supplementary information contains the original uncropped blot images. Simultaneously, released virions were also titrated on TZM-bl cells, briefly equal volumes of cleared supernatants were used to infect TZM-bl cells (1 × 10^4^ cells/well) in triplicates in the presence of DEAE dextran (25 µg/ml). 48 hrs after infection the luciferase activity was measured using britelite plus luciferase assay kit (PerkinElmer, Inc.). Quantification of the protein band intensity was performed using NIH ImageJ software. The virus release efficiency was calculated as the amount of virion-associated p24 as a fraction of the total amount (cell- plus virion-associated) of Gag.

### Replication kinetics

Single and multiple cycle replication assays were performed using TZM-bl and CEM-CCR5 cells respectively. The virus stocks were normalized for RT-activity using Lenti RT activity kit (Cavidi, Sweden).

For single cycle infectivity assay virus stocks normalized for RT-activity (2000 pg/mL) and further serial two fold dilutions were used to infect TZM-bl cells (1 × 10^4^ cells/well) in triplicates in the presence of DEAE dextran (25 µg/ml). 48 h after infection the luciferase activity was measured using britelite plus luciferase assay kit (PerkinElmer, Inc.).

For multiplecycle replication assays CEM-CCR5 cells (1 × 10^5^ cells/well) were infected with equal amount of RT-activity (2000 pg/mL) for each pIndie C1 and pIndie C1-PYRE virus in triplicate in presence of polybrene (1.5 µg/ml) and incubated for 12–16 hours at 37 °C in 5% CO2. After incubation, cells were washed to remove unbound virus. The culture supernatant (Day 0) was collected after centrifugation. Cells were then resuspended in 1 mL of fresh culture medium and incubated at 37 °C in 5% CO2. Subsequently, 0.5 ml of cell-free medium containing viruses was withdrawn for RT-activity measurements at days 2, 4, 8, 10 and 12. The same volumes of fresh medium and new cells were added at each time point. The RT-activity was determined by using Lenti RT activity kit (Cavidi, Sweden).

## Electronic supplementary material

Supplementary Information
