# Supplementary material for: PYRE insertion within HIV-1 subtype C p6-Gag functions as an ALIX-dependent late domain
Source: Sci Rep. 2018 Jun 11;8:8917. doi: 10.1038/s41598-018-27162-1 (PMC5995805; doi:10.1038/s41598-018-27162-1)

Supplementary information

PYRE insertion within HIV-1 subtype C p6-Gag functions as an ALIX-dependent late domain

Devidas Chaturbhuj[1, 2], **Ajit Patil**[1], **and Raman Gangakhedkar**[3,*]

[1] HIV Drug Resistance Laboratory, National AIDS Research Institute (ICMR), Pune, India

[2] Symbiosis International University (SIU), Lavale, Pune, India

[3] Department of Clinical Sciences, National AIDS Research Institute (ICMR), Pune, India

[*] Correspondence to R.G. (email: rgangakhedkar@nariindia.org)

Supplementary Fig. S1 : Uncropped Full-length western blots for Figure. 1b

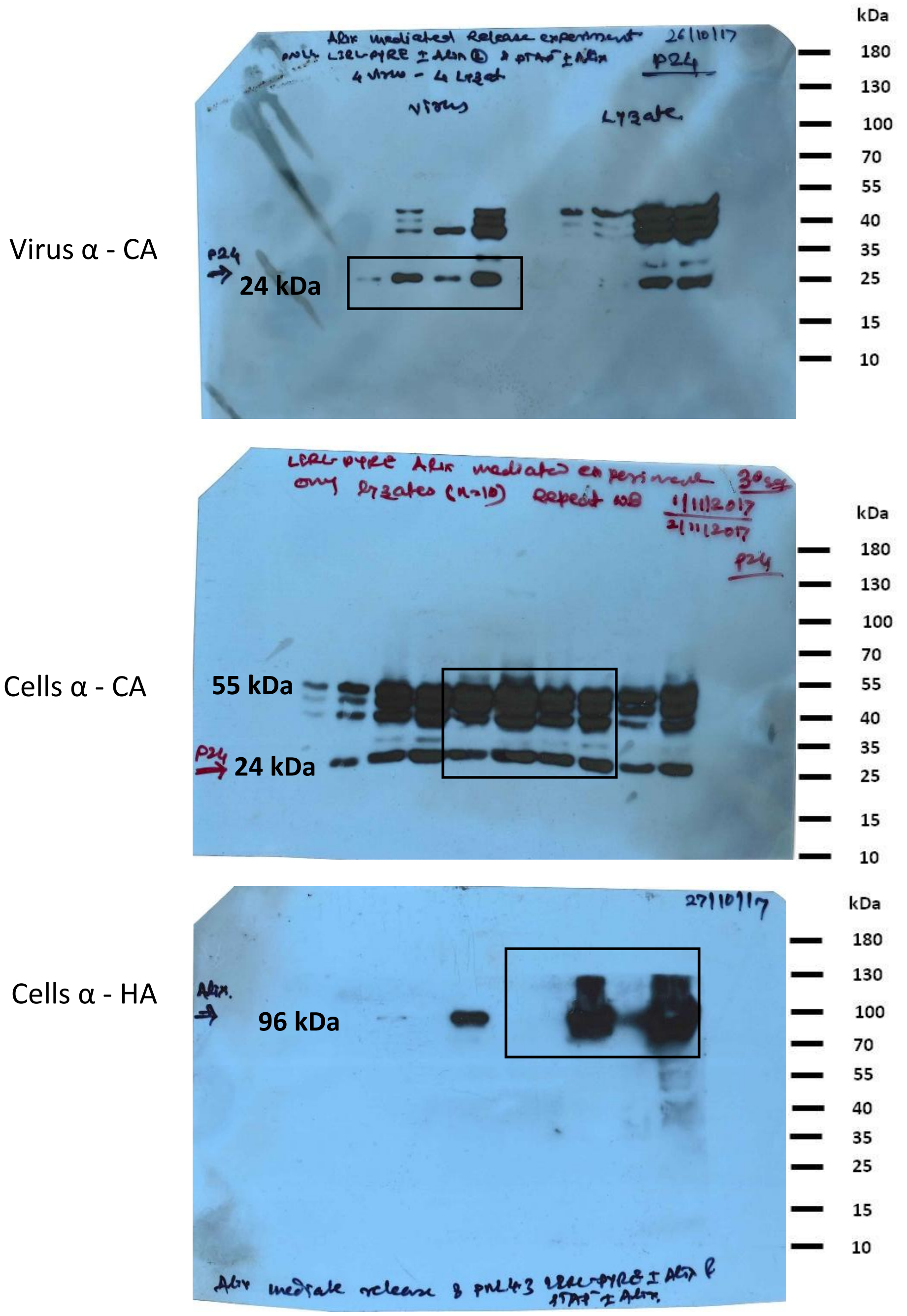

Supplementary Fig . S2 : Uncropped Full-length western blots for Figure. 2

Virus α - CA

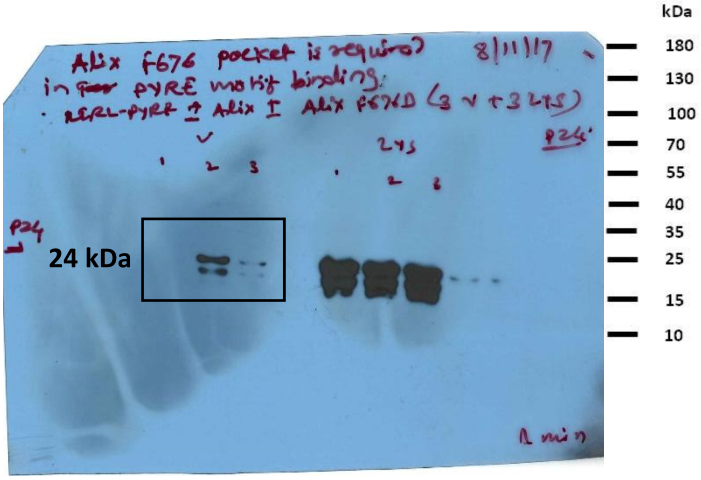

Cells α - CA

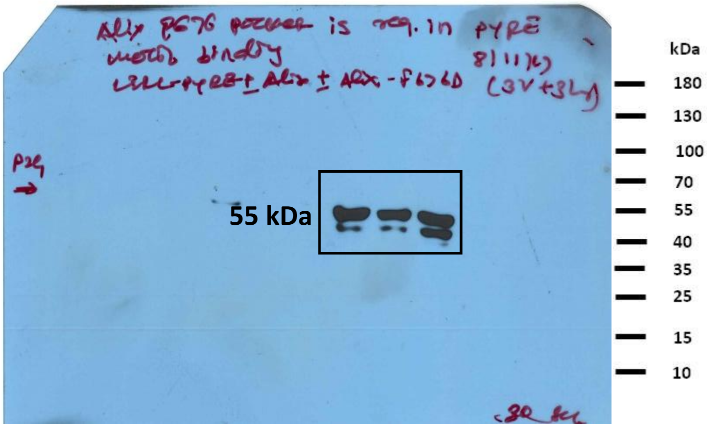

Cells α - HA

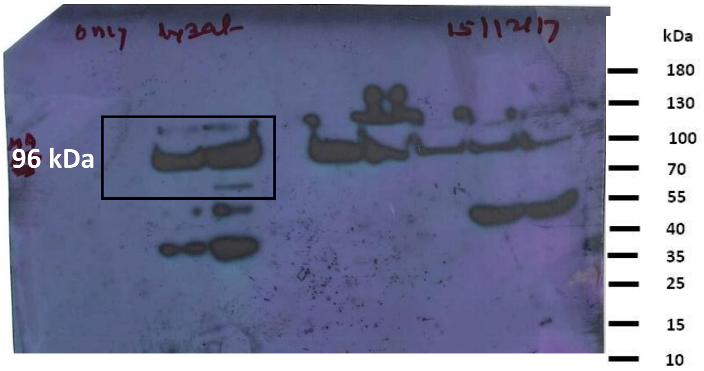

Supplementary Fig . S2a : Uncropped Full-length western blots for Figure. 2a

Supplementary Fig . S2b : Uncropped Full-length western blots for Figure. 2b

Virus α - CA

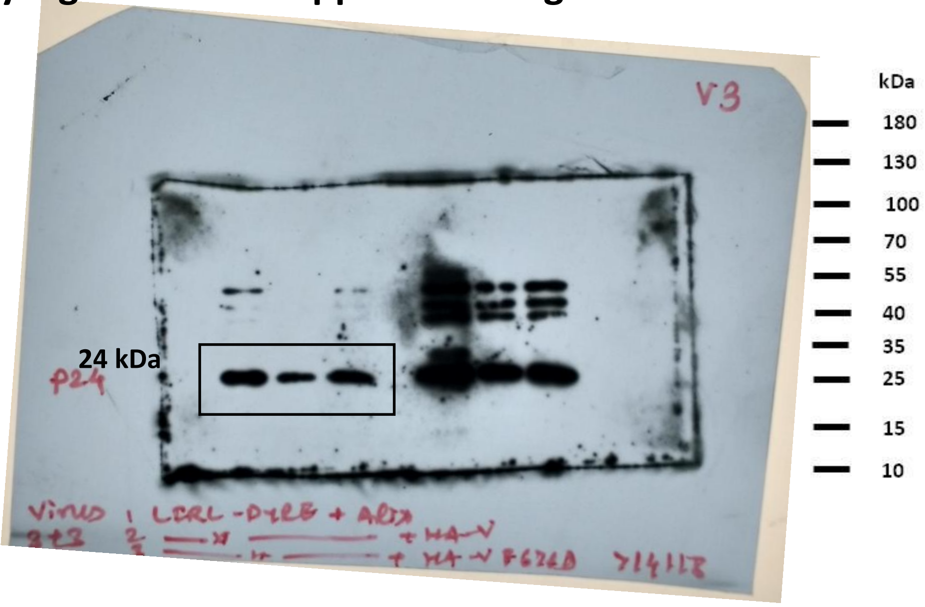

Cells α - CA

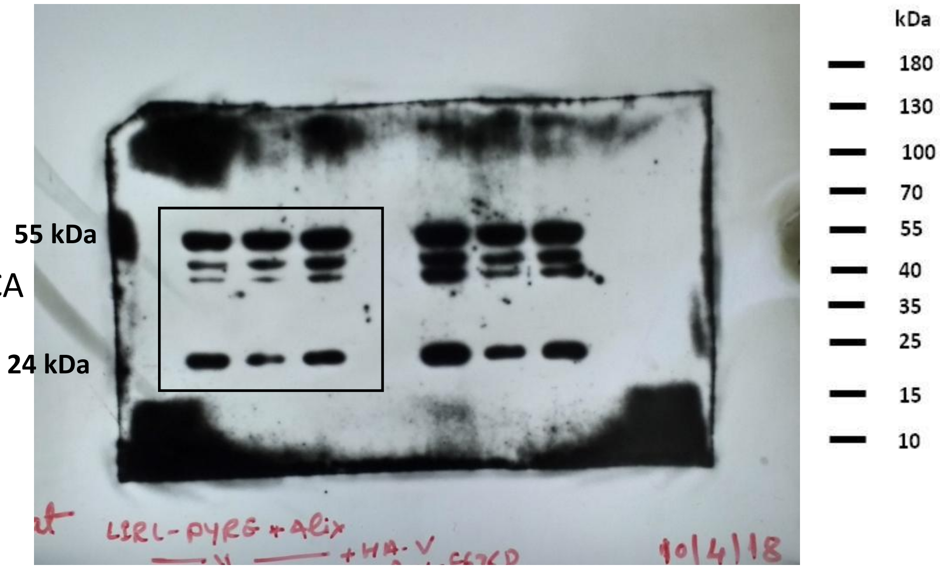

Cells α - HA

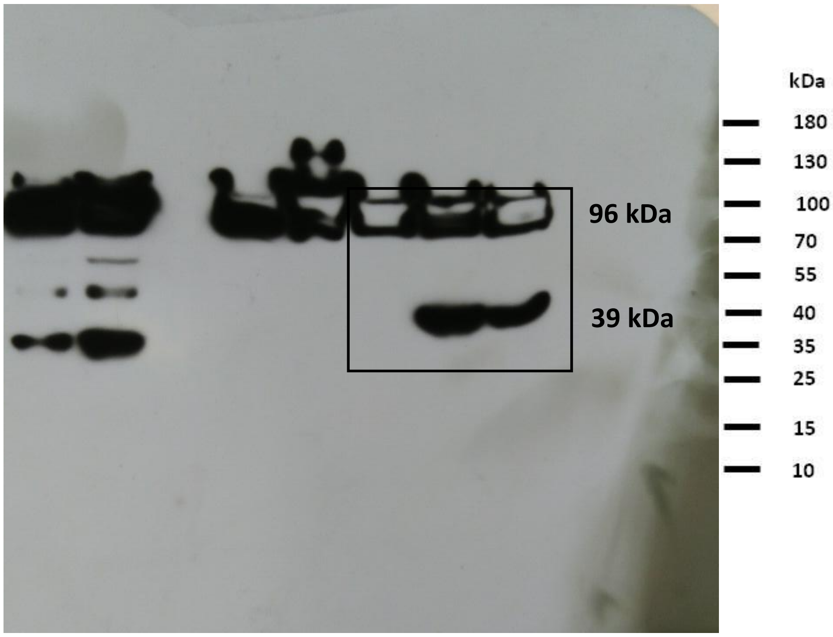

HA-ALIX

HA-V and
HA-VF676D

Supplementary Fig. S3 : Uncropped Full-length western blots for Figure. 3

Virus α - CA

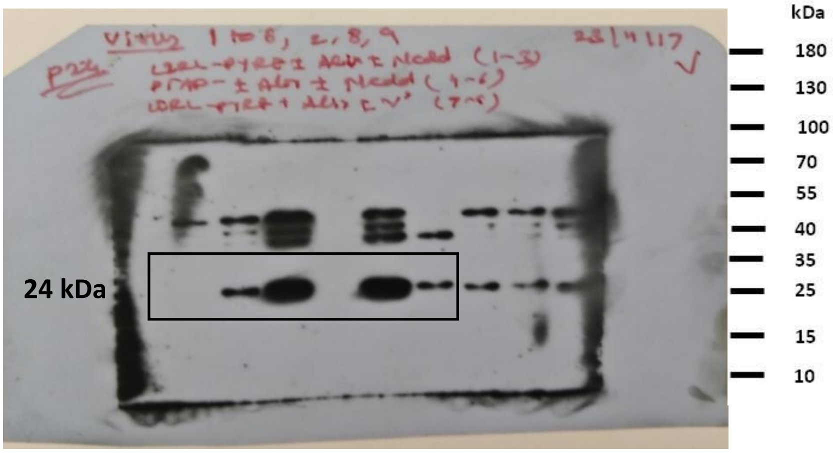

Cells α - CA

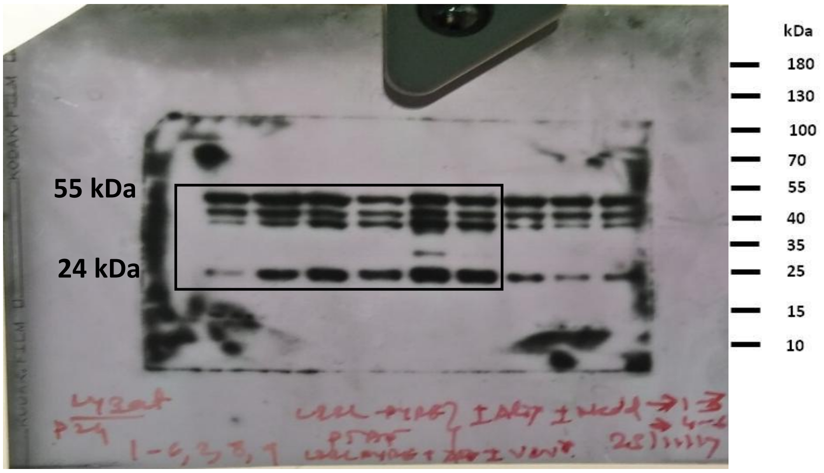

Cells α - HA

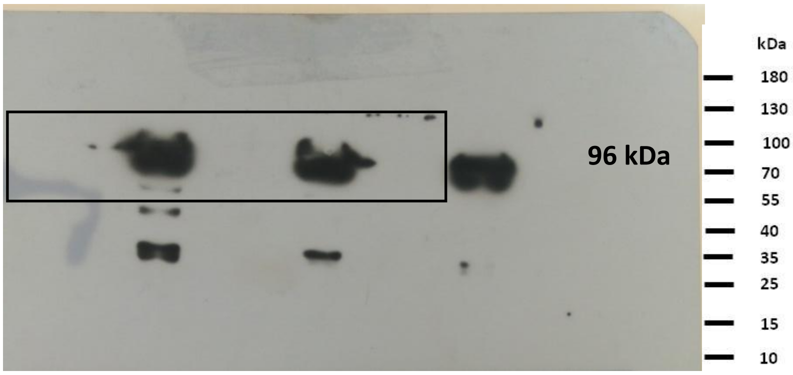

Cells α - FLAG

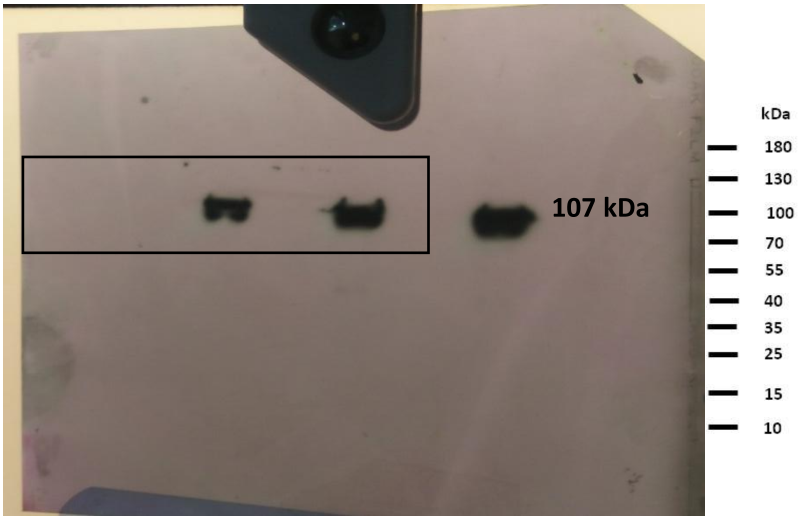

Supplement: Supplementary file 1 — Supplementary Information [file 41598_2018_27162_MOESM1_ESM.pdf]
